# Annexin A1-FPR2/ALX Signaling Axis Regulates Acute Inflammation during Chikungunya Virus Infection

**DOI:** 10.3390/cells11172717

**Published:** 2022-08-31

**Authors:** Simone de Araújo, Victor R. de Melo Costa, Franciele M. Santos, Carla D. Ferreira de Sousa, Thaiane P. Moreira, Matheus R. Gonçalves, Franciel B. Félix, Celso M. Queiroz-Junior, Gabriel H. Campolina-Silva, Maurício Lacerda Nogueira, Michelle A. Sugimoto, Caio S. Bonilha, Mauro Perretti, Danielle G. Souza, Vivian V. Costa, Mauro M. Teixeira

**Affiliations:** 1 Graduate Program in Biological Sciences Physiology and Pharmacology, Department of Physiology and Biophysics, Institute of Biological Sciences, Federal University of Minas Gerais, Belo Horizonte 31270-901, Brazil; 2 Drug Research and Development Center, Institute of Biological Sciences, Federal University of Minas Gerais, Belo Horizonte 31270-901, Brazil; 3 Department of Obstetrics, Gynecology and Reproduction, CHU de Quebec Research Center (CHUL), Université Laval, Quebec, QC G1V 0A6, Canada; 4 Department of Dermatological, Infections, and Parasitic Diseases, School of Medicine (FAMERP), São José do Rio Preto, São Paulo 15090-000, Brazil; 5 Barts and The London School of Medicine and Dentistry, William Harvey Research Institute, Queen Mary University of London, London E1 4NS, UK; 6 Center for Research on Inflammatory Diseases, University of São Paulo, São Paulo 05508-000, Brazil; 7 Institute of Infection, Immunity and Inflammation, University of Glasgow, Glasgow G12 8QQ, UK; 8 Centre for Inflammation and Therapeutic Innovation, Queen Mary University of London, London E1 4NS, UK; 9 Graduate Program in Microbiology, Department of Microbiology, Institute of Biological Sciences, Federal University of Minas Gerais, Belo Horizonte 31270-901, Brazil; 10 Graduate Program in Cell Biology, Department of Morphology, Federal University of Minas Gerais, Belo Horizonte 31270-901, Brazil; 11 Department of Biochemistry and Immunology, Institute of Biological Sciences, Federal University of Minas Gerais, Belo Horizonte 31270-901, Brazil

**Keywords:** CHIKV, Annexin-A1, *FPR2*, neutrophils, Ac_2–26_ peptide

## Abstract

Chikungunya (CHIKV) is an arthritogenic alphavirus that causes a self-limiting disease usually accompanied by joint pain and/or polyarthralgia with disabling characteristics. Immune responses developed during the acute phase of CHIKV infection determine the rate of disease progression and resolution. Annexin A1 (*AnxA1*) is involved in both initiating inflammation and preventing over-response, being essential for a balanced end of inflammation. In this study, we investigated the role of the *AnxA1-FPR2/ALX* pathway during CHIKV infection. Genetic deletion of *AnxA1* or its receptor enhanced inflammatory responses driven by CHIKV. These knockout mice showed increased neutrophil accumulation and augmented tissue damage at the site of infection compared with control mice. Conversely, treatment of wild-type animals with the *AnxA1* mimetic peptide (Ac_2–26_) reduced neutrophil accumulation, decreased local concentration of inflammatory mediators and diminished mechanical hypernociception and paw edema induced by CHIKV-infection. Alterations in viral load were mild both in genetic deletion or with treatment. Combined, our data suggest that the *AnxA1-FPR2/ALX* pathway is a potential therapeutic strategy to control CHIKV-induced acute inflammation and polyarthralgia.

## 1. Introduction

Chikungunya virus (CHIKV) is a re-emerging arbovirus that causes a disease known as chikungunya fever (CF). This disease is characterized by intense acute inflammation, and it is accompanied by several symptoms, including fever, skin rash, muscle pain, and severe joint pain (polyarthralgia) [1,2]. Acute CHIKV infection induces robust innate and adaptive immune responses characterized by a high systemic production of inflammatory mediators, including IL-6 and IL-1β [3,4]. Furthermore, CD4 and CD8 T cells are mainly involved in joint damage seen in CHIKV disease [2]. At the same time, innate immune cells such as neutrophils and monocytes are recruited to the infection site [5,6]. Neutrophils produce reactive oxygen species (ROS) and neutrophil extracellular traps (NET) which contribute to a process of controlling CHIKV acute infection [7,8]. However, persistent inflammation can lead to the chronic phase of CF with the development of incapacitating rheumatic disorders [9,10]. These disorders are known as chronic chikungunya arthritis and may last for several months or years [11]. Sometimes the pain associated with this illness can be so intense that it disrupts lifestyle, work and personal life, hampering the ability to perform simple activities [2,12]. Curiously, chronic diseases are characterized by impairment of natural resolution mechanisms, as already demonstrated in the literature [13,14,15,16].

CHIKV has become a serious public health issue in the last two decades [11,17]. Nevertheless, treatment remains primarily supportive since no antiviral drug is available. Management includes administration of non-steroidal anti-inflammatory drugs, other analgesic medication, rehydration, and rest [2,13]. We have argued that understanding mechanisms involved in controlling acute inflammation may lead to the development of new therapeutic strategies to treat viral infections, including that caused by CHIKV [18,19].

Pro-resolving mediators are mediators that play pivotal roles in the vascular response and leukocyte trafficking, from initiation to resolution [16,20]. Annexin A1 (*AnxA1*) is an anti-inflammatory and pro-resolving mediator that belongs to a group of Ca^2±^ dependent phospholipid-binding proteins [21]. These proteins plays a role in various cellular functions, including inflammatory responses, proliferation, differentiation, and apoptosis [22,23]. *AnxA1* is expressed in a variety of immune cells such as neutrophils, monocytes, macrophages, and mast cells [24,25,26]. Then, by acting on the Formyl-peptide receptor 2 (*FPR2*)/*ALXR*, *AnxA1* is responsible for regulating neutrophil migration to inflammatory sites, inducing neutrophil apoptosis and decreasing inflammation and pain in different experimental models [27,28,29,30,31,32].

CHIKV infection is characterized by chronic inflammation with high probability of long-term pain [4,11]. However, *AnxA1* is essential for regulating excess inflammation, and it is also important for promoting complete resolution of the inflammatory process [23,33]. Evidence shows that this protein may be defective or at lower levels in some patients infected with the virus [34]. Therefore, it is likely that failure in resolutive mechanisms, as shown in dengue and Zika, also contributes to the severeness of CHIKV [32,35]. Here we hypothesize that *AnxA1* is involved in the control mechanism of acute inflammation during CHIKV infection. Thus, wild-type and *AnxA1* KO and its receptor *Fpr2/3* (*FPR2/ALX* in humans) deficient mice strains were used to investigate the relevance of this protein. Next, we evaluated the therapeutic effects of Ac_2–26_ (mimetic peptide *AnxA1*) in a murine model of CHIKV infection. Our study provides the first evidence that the *AnxA1–FPR2/ALX* pathway is an important regulator of the acute inflammatory response, with the possibility to reduce pain during CHIKV infection.

## 2. Materials and Methods

### 2.1. Experimental Model

3–4 week-old BALB/c and C57BL/6, *AnxA1*-deficient mice (BALB/c background) and *Fpr2/3-*deficient mice (C57BL/6 background) were originally generated as described in [36,37], and bred in-house (Bioterium of the Federal University of Minas Gerais, Belo Horizonte, Brazil). The mice were kept under specific pathogen-free conditions at a constant temperature (25 °C) with free access to chow and water in a 12 h light/dark cycle. The mice were inoculated subcutaneously in the right rear footpad with 10^6^ PFU of CHIKV in PBS (30 μL volume). They were then evaluated by haematological parameters, hypernociception, and paw edema from day zero to 21 or 28 days after infection. Mechanical hypernociception was evaluated with a modified electronic pressure measurement test for mice (electronic Von Frey), as previously described [38]. The results were expressed as the force (g) required to induce dorsiflexion of the tibiotarsal joint followed by paw withdrawal. Foot swelling, an indicator of local inflammation, was recorded by a digital vernier caliper and the increment of the edema index (Δ) was defined as the difference in basal values (day 0) and the post-infection measurement, expressed in mm. The mice were euthanized at 1, 3, 7, or 14 days post-infection (dpi). Samples of footpad, ankle, knee, popliteal lymph node (pLN), quadriceps, spleen, liver, or plasma were evaluated to determine viral loads by plaque assay. The footpads were also analyzed by ELISA, histology, and flow cytometry. This study was approved by the Ethics Committee in the Use of Animals (CEUA) of the UFMG (protocol number 135/2019).

### 2.2. Virus

The CHIKV strain is a human isolate (genotype ECSA–strain BHI3762, accession number H804917), kindly provided by Dr. Maurício Lacerda Nogueira, from the Medical School of University of São José do Rio Preto–FAMERP. CHIKV stocks were produced in African green monkey kidney Vero cells (CCL-81) from the Cell Bank of Rio de Janeiro, CBRJ (Duque de Caxias, Brazil). Briefly, Vero cells were grown in Dulbecco’s Modified Eagle’s Medium (DMEM; Cultilab, Campinas, Brazil), supplemented with 10% inactivated fetal bovine serum (FBS; Cultilab, Brazil) and 1% Penicillin/Streptamycin/Glutamine (GIBCO) and kept in a humidified incubator at 37 °C with 5% CO_2_ atmosphere for four days. The cell supernatant was collected, centrifuged (3000× *g* for 10 min) and then concentrated using a Vivacell 100 centrifugal concentrator (Sartorius, Göttingen, Germany). The Vero cells were plated in 24-well plates and infected with 10-fold serial dilutions to obtain the viral titer. The plates were incubated for 72 h (37 °C with 5% CO_2_ atmosphere), fixed with 10% formaldehyde, and stained with 1% violet crystal.

### 2.3. Viral Load Measurement

Viral replication and titration by plaque-forming units (PFU) assays were performed using Vero cells obtained from the CBRJ as described above. The results are presented as the log of PFU per/g of tissue or PFU per/mL of plasma.

### 2.4. Measurement of Inflammatory Mediators

Myeloperoxidase (MPO) levels were measured as previously described [29]. Cytokine (IL-1β, IL-6, TNFα, and IL-10), and chemokine (CXCL1, CXCL2, and CCL2) levels were evaluated by ELISA commercial kits according to the manufacturer’s instructions (R&D Systems, Minneapolis, MN, USA). The results are expressed in picograms (mean ± error) normalized to 100 mg of tissue.

### 2.5. Plasma Levels of AnxA1

Blood was collected in heparinized tubes and centrifuged at 5000× *g* for 10 min at room temperature. Plasma was collected and stored at −80 °C until assayed [32]. *AnxA1* levels in plasma samples obtained from BALBc or C57BL/6 mice along with the kinetics of infection with CHIKV (10^6^ PFU) were measured by ELISA. A specific LSBio kit (LifeSpan BioSciences, Inc, Seattle, WA, United States) was used, following the manufacturer’s recommendations. The results are expressed as ng per ml of plasma.

### 2.6. Flow Cytometry

Paws were collected from *AnxA1* KO and WT (BALB/c) mice 2- or 7-days post-CHIKV infection. The tissue was homogenized, and the cells were digested with 1 mg/mL collagenase IV (Sigma-Aldrich, St. Louis, MI, USA) in complete DMEM for 60 min at 37 °C. The cells were then counted in a Neubauer chamber and stained as previously described [39]. The cells were acquired using a FACS CantoTM II (BD Biosciences, San Jose, CA, USA) and analyzed with FlowJo v10.7.1 https://www.flowjo.com/solutions/flowjo/downloads (Tree Star, Inc., Ashland, OR, USA) (accessed on 11 February 2021).

### 2.7. Histology and Immunohistochemistry

The paw tissues were removed and fixed in 10% *v*/*v* buffered formalin for 48 h, decalcified in EDTA 14%, dehydrated in graded ethanol, and embedded in paraffin. Sections of 5 μm were stained with Hematoxylin-Eosin (H&E) before analysis. The intensity and extension of inflammatory infiltrate, and loss of muscle tissue architecture were evaluated according to the following cumulative score system: absence of lesion (0), mild lesion (1 and 2), moderate lesion (3 and 4), intense lesion (5 and 6), and very intense lesion (7). The sections were incubated with anti-*AnxA1* (1:400; Invitrogen, Thermo Fisher Scientific, Waltham, MA, USA), followed by a biotinylated secondary antibody, and the color was developed using DAB (3,3′-diaminobenzidine) (Sigma, Sigma-Aldrich). The sections were counterstained with hematoxylin, mounted, and examined using a Motic microscope (Carl Zeiss, Gottingen, Germany).

### 2.8. Ac_2–26_ Treatments

Ac_2–26_, a synthetic derivative corresponding to aa 2–26 of the N-terminal region of *AnxA1*, was reconstituted in DMSO. The following dissolution was made in sterile PBS (final concentration of 2% DMSO), as previously described [32]. Prophylactic or therapeutic Ac_2–26_ treatments were given to infected mice or uninfected controls. In the prophylactic approach, mice were given, intraperitoneally, 150 µg of the drug, daily, about 1 h before infection and the treatment was continued for the next three days post-infection. In the therapeutic approach, mice were given Ac_2–26_ (150 µg/mice) in a single dose 24 h post-infection.

### 2.9. Statistical Analysis

The Shapiro–Wilk test was applied to determine data normality. All results were presented as the mean ± SEM. Differences between groups were evaluated using analysis of variance (ANOVA) or a two-way ANOVA, followed by Tukey or Sidak post hoc analysis, respectively. Data from histology, and *AnxA1* expression detected by immunohistochemistry were statistically examined by Kruskal–Wallis with Dunn’s test (non-parametric) or the Mann–Whitney test. The calculations were performed using GraphPad Prism 8.0 (San Diego, CA, USA) (accessed on 19 July 2022). As indicated in the figure legends, the significance level was set at *p* < 0.05 for all analyses.

## 3. Results

### 3.1. CHIKV Infection Does Not Alter AnxA1 Plasma Levels but Increases Its Expression at the Site of Virus Inoculation

**Figure 1:**
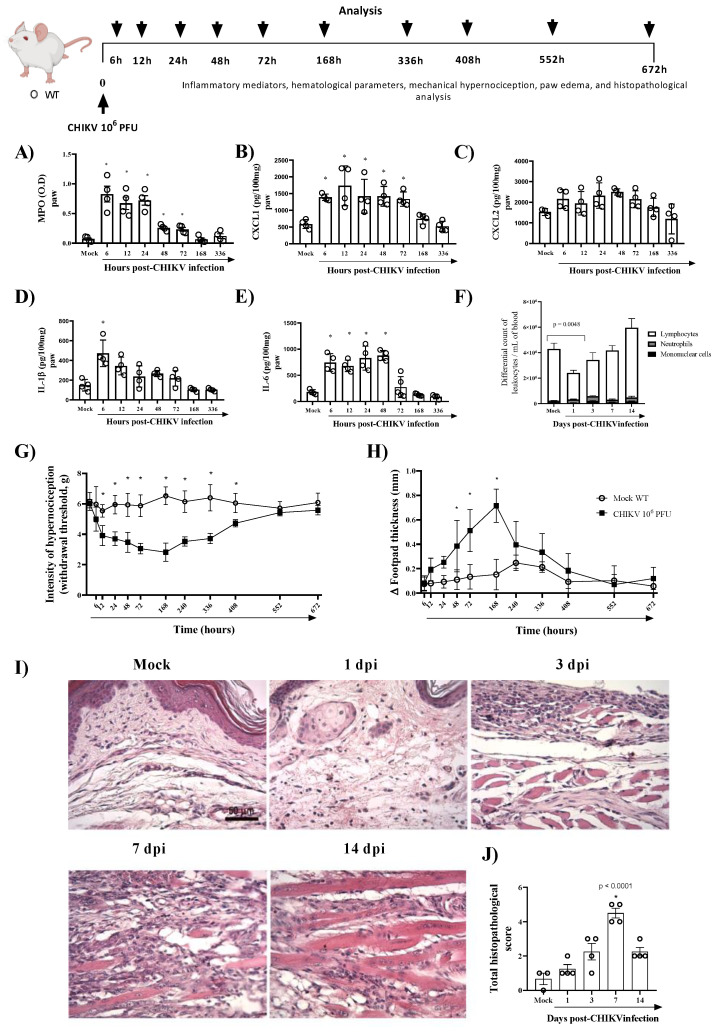
CHIKV-infected mice show inflammation, prolonged hypernociception, edema and tissue damage in the paw. Wild-type (WT/BALBc) mice were infected by the intraplantar (i.pl.) route with CHIKV 10^6^ PFU and then were euthanized at 6, 12, 24 (1 dpi)-, 72 (3 dpi)-, 168 (7 dpi)- or 336 (14 dpi)-hours post-infection. (**A**) MPO, (**B**) CXCL1, (**C**) CXCL2, (**D**) IL-1β, and (**E**) I IL-6 levels in the footpad of mice. (**F**) Differential white blood cell count. The cytokines and chemokines levels were measured by ELISA and were shown as pg per 100 mg of the footpad. (**G**) Mechanical hypernociception and (**H**) Paw swelling of mock (*n* = 5) and WT + CHIKV (*n* = 5) groups. Data for mechanical hypernociception are shown as the force (g) required to induce dorsiflexion of the tibiotarsal joint, followed by paw withdrawal. The results for paw edema are expressed by Δ of the difference between the baseline and post-infection measurements, expressed in mm. Statistical analysis was performed using a two-way ANOVA with Sidak comparisons tests. Data are presented as mean ± SEM (*n* = 5–6 per group); * *p* < 0.05 when compared to control uninfected mice (mock). (**I**) Representative H&E images (magnification 400×, scale 50 µm) of inflamed joint footpad at 1, 3, 7 or 14 days post-infection. (**J**) Histopathological scoring (*n* = 3–4). Statistical analysis was performed using Kruskal–Wallis with Dunn’s multiple comparisons. * for *p* < 0.05 when compared with the WT infected group.

To study CHIKV-induced pathogenesis, we developed an experimental model in immunocompetent mice with similar clinical signs found in infected patients, including hyperalgesia, edema, and intense production of pro-inflammatory cytokines. As seen in Figure 1A, the infection with 10^6^ PFU of CHIKV induced polymorphonuclear neutrophils (PMN) accumulation, as assessed by measuring tissue levels of MPO, from 6 to 72 h post-infection (hpi). Similarly (except for CXCL2), CXCL1, IL-1β and IL-6 levels were significantly increased in BALB/c mice infected with CHIKV (Figure 1B–E). IL-1β peaked 6 hpi and reduced after that, while IL-6 and CXCL1 levels remained elevated up to 48 hpi or 72 hpi, respectively (Figure 1B,D,E). The hematological parameters showed that infection with 10^6^ PFU of CHIKV induced leukopenia on the first day post-infection (Figure 1F). In the current study, intraplantar injection of CHIKV induced articular hypernociception from 12 hpi with persistence up to 17 days post infection (dpi). At 21 dpi, hypernociception was resolved entirely, and the animals returned to basal levels (similar to the control group) (Figure 1G). From 48 hpi onwards, edema formation began in the paw, which peaked 7 days after infection, reducing after that period (Figure 1H). Histopathological analysis of the paw revealed that CHIKV infection induced an inflammatory reaction with damage to tissue architecture that was more severe at 7 dpi (Figure 1I,J). Subsequently, we demonstrated that CHIKV inoculation results in a self-limited disease in mice with production of inflammatory mediators and prolonged hyperalgesia similar to chikungunya seen in humans.

**Figure 2:**
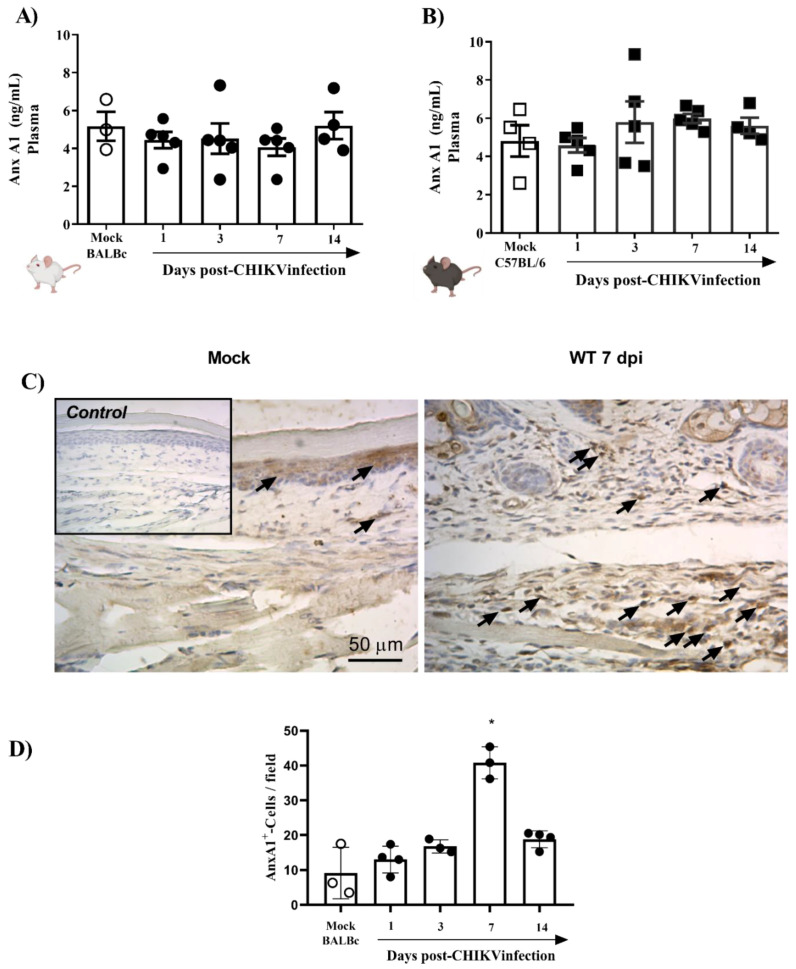
*AnxA1* is expressed in inflamed paw tissues of CHIKV-infected mice. BALB/c or C57BL/6 mice were infected through the intraplantar (i.pl.) route with CHIKV 10^6^ PFU and were euthanized at 1, 3, 7 or 14 days post-infection. Paws were collected and processed for analysis of *AnxA1* expression by immunohistochemistry (IHC), and the plasma was used to measure *AnxA1* levels. (**A**) *AnxA1* levels in the plasma of BALB/c mice, (**B**) *AnxA1* levels in the plasma of C57BL/6 mice. Results are represented as ng of *AnxA1* per mL of plasma. (**C**,**D**) Score and representative *AnxA1* IHC images of an inflamed joint footpad. IHCs were quantified as the average of positive cells in five independent fields per tissue at 400×. Statistical analysis was performed using the Kruskal–Wallis with Dunn’s comparisons test. Data were presented as mean ± SEM (number of animals is shown in each graph); * *p* < 0.05 when compared with control uninfected mice (mock).

To verify whether the systemic levels of *AnxA1* were altered during the infection, mice plasma from two different strains (BALB/c or C57BL/6), infected or not with 10^6^ PFU of CHIKV, were analyzed. The results demonstrated that plasma levels of *AnxA1* were not altered in CHIKV-infected BALB/c (Figure 2A) and C57BL/6 mice (Figure 2B). Subsequently, we evaluated whether the immunostaining of *AnxA1* was altered in hind paws. For this purpose, BALB/c mice were euthanized 1, 3, 7 or 14 days after infection with CHIKV and the paws were processed and evaluated by immuno-histochemistry. The results show that at 7 dpi there was a significant increase in the immunostaining of *AnxA1* in the footpad sections compared with the uninfected control (Figure 2C,D). Furthermore, cells containing *AnxA1* were observed throughout the epidermis, i.e., keratinocytes, and in the connective tissue cells, mainly infiltrated leukocytes, in WT (BALB/c) mice after infection with CHIKV. Combined, these results suggest that local inflammation triggered by the CHIKV infection is attended by elevated local levels of *AnxA1*, while circulating *AnxA1* remains constant. Thus, the local increase in *AnxA1* expression during CHIKV infection motivated the investigation into the role of the *AnxA1-FPR2/ALX* pathway in CHIKV disease.

### 3.2. Mice Lacking AnxA1 or the Fpr2/3 Receptor Exhibit Exacerbated Inflammation and Greater Tissue Damage at Site of Infection

**Figure 3:**
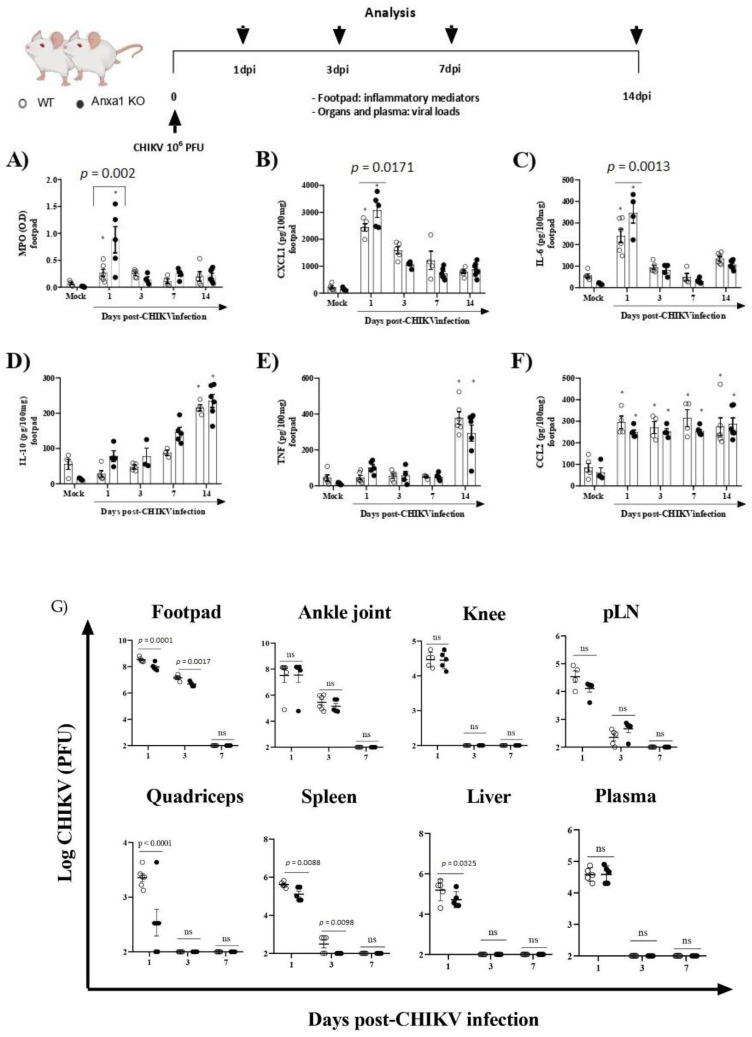
Absence of Annexin A1 (*AnxA1*) increases inflammatory response induced by CHIKV. Annexin-A1 (*AnxA1* KO) or WT (BALB/c) mice were infected by the intraplantar (i.pl.) route with CHIKV 10^6^ PFU and then were euthanized at 1, 3, 7 or 14 days post-infection. (**A**) MPO, (**B**) CXCL1, (**C**) IL-6, (**D**) IL-10, (**E**) TNF, and (**F**) CCL2 levels in the footpad of mice. The cytokines and chemokines levels were measured by ELISA and are shown as pg per 100 mg of footpad. (**G**) Plasma and organs of animals (including footpad, ankle joint, knee, pLN, quadriceps, spleen, and liver) were extracted at 1, 3, 7 or 14 days post-infection and assessed for viral titers using plaque assays on Vero CCL-81 cells. Results are shown as the number of PFU per mL plasma or mg tissue. Statistical analysis was performed using the two-way ANOVA with Sidak comparisons test. Data are presented as mean ± SEM (*n* = 5–6 per group); * *p* < 0.05 when compared with control uninfected mice (mock). pLN: popliteal lymph node.

To investigate the role of the *AnxA1-FPR2/ALX* pathway in CHIKV infection, we infected animals lacking *AnxA1* or its receptor. MPO levels in the footpad were significantly increased in *AnxA1* KO (at 1 dpi) in comparison to their respective WT (BALB/c) controls (Figure 3A). Likewise, the neutrophil accumulation within the tissue was followed by enhanced levels of CXCL1 and IL-6 in the paw of *AnxA1*-deficient mice at 1 dpi (Figure 3B,C). We found IL-10 and TNF levels were augmented, followed by a sustained increase in CCL2 levels in the footpad in either *AnxA1* KO or WT (BALB/c) animals compared with their respective uninfected controls (Figure 3D–F). Augmented TNF has been implicated in CHIKV-induced chronic arthritis and related to the persistence of pain [40]. Moreover, CCL2 is involved in cellular infiltration during CHIKV infection [6,41]. At 1 and 3 dpi, a minimal but significant reduction was observed in viral titers in the footpad, quadriceps, spleen, and liver of *AnxA1* KO compared with WT (BALB/c) animals (Figure 3G).

**Figure 4:**
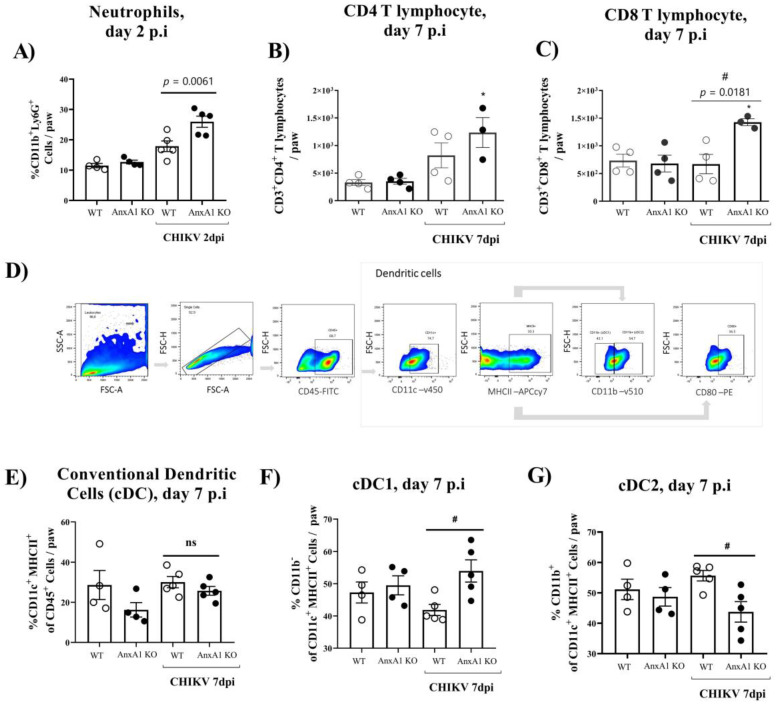
Immune cell accumulation in the paw of *AnxA1* KO or WT mice infected with CHIKV. (**A**) Percentage of neutrophils, (**B**) CD4^+^ T cells, (**C**) CD8^+^ T cells, (**D**) gate strategy, (**E**) Conventional dendritic cells (cDC), (**F**) cDC1, (**G**) cDC2, per footpad at 2 or 7 dpi. Statistical significance was calculated using one-way ANOVA with Tukey comparisons test. Data are presented as mean ± SEM (*n* = 4–5 per group); * *p* < 0.05. when compared with the uninfected control (mock) or ^#^ *p* < 0.05 when compared with *AnxA1* KO and WT infected mice.

**Figure 5:**
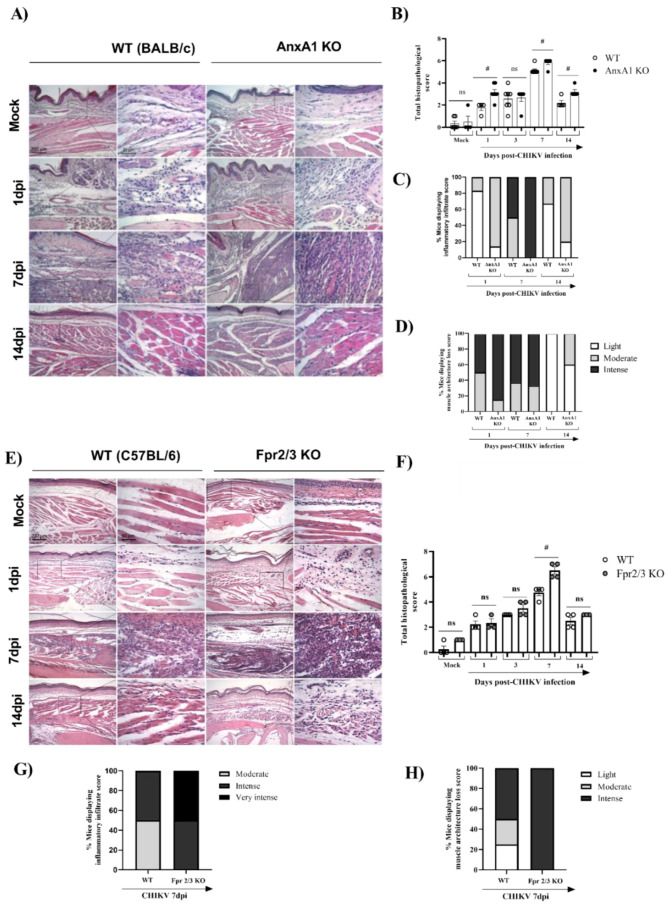
Absence of *AnxA1* or *FPR2* impairs tissue integrity and increases the inflammatory response in the paw of mice infected with CHIKV. *AnxA1* KO or WT (BALB/c), and *FPR2* (*Fpr2/3* KO) or WT (C57BL/6) mice were infected with CHIKV (10^6^ PFU/30μL, i.pl.) and then were euthanized at 1, 3, 7 or 14 days post-infection. (**A**,**E**) Representative H&E images (magnification 100× or 400×. Scales: 200 µm at low magnification and 50 µm at high magnification) of inflamed joint footpads of mice. (**B**,**F**) Total histopathological scoring of inflammatory infiltrate, and tissue damage in the joint footpad. (**C**,**D**,**G**,**H**) show the percentage of mice presenting the score of inflammatory infiltrate or loss of muscle architecture in paw. Data are representative of two independent experiments, (*n* = 3–4). Statistical analysis was performed using the Mann–Whitney test. The *AnxA1* KO or *Fpr2/3* KO group was considered statistically significant from the WT group when # *p* < 0.05.

We subsequently decided to perform an in-depth characterization of the immune cells during the course of infection. For this purpose, we used the paw or pLN. The pLN is the nearest lymph node to the CHIKV infection site in the footpad. CHIKV infection improved the number of CD45^+^ cells in the paw in both WT (BALB/c) and *AnxA1* KO animals in comparison with uninfected controls at 2 dpi (Supplementary Figure S1A). This phenotype was maintained at 7 dpi. However, a significant decrease in the percentage of macrophages with an M2 profile was observed in the paws of infected animals compared with the uninfected controls (Supplementary Figure S1D). Analysis of immune cells in the pLN 2 dpi showed that *AnxA1* deficiency did not cause alterations in the percentage of CD4^+^ or CD8^+^ T lymphocytes or in their activation profile (Supplementary Figure S1F–I). Interestingly, in the absence of *AnxA1*, the animals showed a significant increase in neutrophils (CD11b^+^Ly6G^+^) at 2 dpi (Figure 4A), followed by an increase at 7 dpi in the count of CD8 T lymphocytes (CD3^+^CD8^+^) (Figure 4C), and conventional dendritic cells (cDCs1) (CD11c^high^^+^MHCII^+^CD11b^−^) (Figure 4F), in addition to a decrease in cDCs2 cells (CD11c^high^^+^MHCII^+^CD11b^+^) (Figure 4G) in the paws compared with infected WT (BALB/c) animals. Histopathological analysis at 1, 7, or 14 days post-infection revealed a significant increase in tissue injury and showed that paws of *AnxA1* KO mice had greater tissue inflammation compared with WT (BALB/c) mice, peaking at 7 dpi (Figure 5A,B), indicating a protective role of *AnxA1* in CHIKV disease.

Finally, we investigated whether the absence of *FPR2*, the receptor for *AnxA1*, would impact CHIKV-induced inflammation. For this, we used *Fpr2/3* KO mice, a good knockdown model of human *FPR2/ALX* [38]. Similar to *AnxA1* KO infected mice, MPO levels in the footpad were significantly increased in *Fpr2/3* KO (at 3 dpi) compared with WT (C57BL/6) animals (Supplementary Figure S2A), suggesting that these effects are due to disruption of the *AnxA1-FPR2/ALX pathway*. We also verified a minimal reduction in viral load in the spleen and liver of *Fpr2/3* KO mice compared with their counterparts, as seen in Supplementary Figure S2C–H. Curiously, animals deficient in the *AnxA1* receptor showed increased hypernociception at 21 dpi (Supplementary Figure S3C,D). In agreement with the higher PMN accumulation observed in *Fpr2/3* KO mice, histological analyses of paws showed intense damage in these mice, whereas WT (C57BL/6) animals had mild to moderate damage progression. These findings were more evident at 7 dpi (Figure 5C,D). Taken together, these results indicate that the absence of the *AnxA1-FPR2/ALX* signaling pathway exacerbates inflammation at the virus inoculation site and increases tissue damage during CHIKV infection. This suggests the relevance of *AnxA1* in local inflammation control.

### 3.3. Treatment with Exogenous AnxA1 (Ac_2–26_ Peptide) Reduces Inflammation and Mechanical Hypernociception Induced by CHIKV

**Figure 6:**
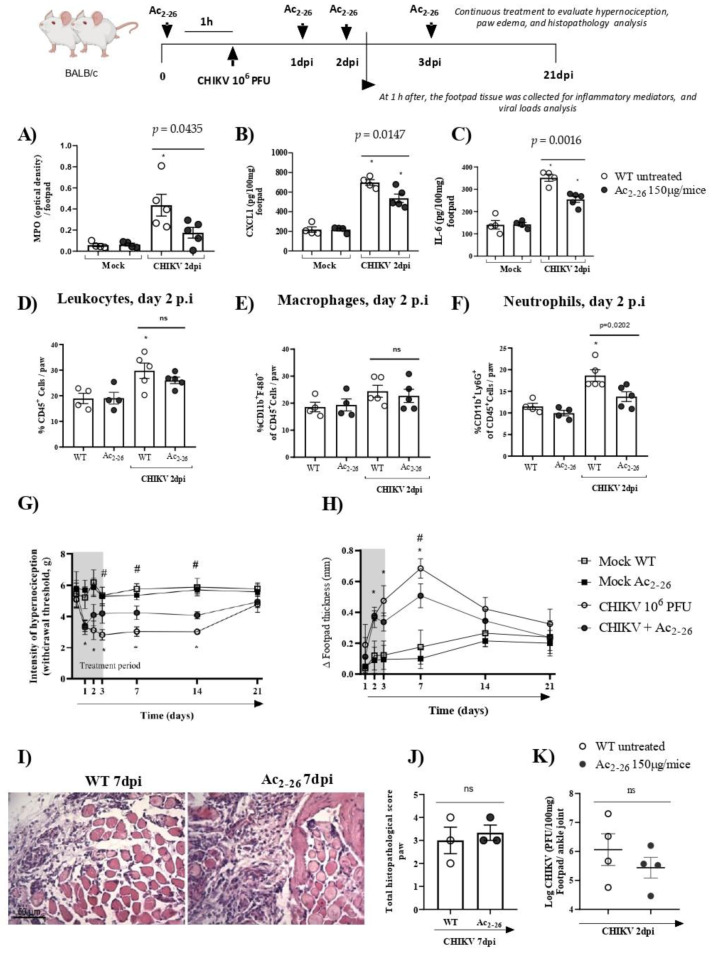
Effects of Ac_2–26_ treatment in inflammatory responses and hypernociception induced by CHIKV-infection. BALB/c mice received Ac_2–26_ (150 µg/mice, intraperitoneal route), and 1 h after treatment were infected with CHIKV (10^6^ PFU/30μL, i.pl.). The treatment was continued with daily doses at 24, 48, or 72 h post-infection, equivalent to 1, 2, and 3 days post-infection, respectively. At 1 h after the 48 h treatment (2 dpi), the footpad tissue was collected. (**A**) MPO, (**B**) CXCL1, and (**C**) IL-6 levels in the footpad. (**D**) Percentage of CD45^+^ cells, (**E**) macrophages, and (**F**) neutrophils per footpad in mock, CHIKV-infected (WT + CHIKV), and WT + CHIKV + Ac_2–26_ mice at 2 dpi. (**A**–**F**) Data were analyzed by one-way ANOVA with Tukey post-test, * *p* < 0.05. (**G**) Mechanical hypernociception was evaluated by an electronic pressure-meter at 1, 2, 3, 7 or 21 days after the infection treated or not with Ac_2–26_. (**H**) Paw swelling of WT + CHIKV and WT + CHIKV + Ac_2–26_ groups. Data for mechanical hypernociception were shown as the force (g) required to induce dorsi-flexion of the tibiotarsal joint, followed by paw withdrawal. Results for paw edema are expressed by Δ of the difference between the baseline measurement and the post-infection measurement, expressed in mm. Statistical significance was calculated using two-way ANOVA with Sidak comparisons test. Data are presented as mean ± SEM (*n* = 4–5 per group); * *p* < 0.05. Representative results of two experiments performed independently. # *p* < 0.05 when compared with *AnxA1* KO and WT infected mice. (**I**) Representative H&E images of inflamed joint footpad on 7 dpi. (**J**) Histopathological scoring. Statistical analysis was performed using the Mann–Whitney test, (*n* = 3 per gr–up), ns–not significant. (**K**) Viral loads recovered from ankle joint of infected mice treated or not with Ac_2–26_, examined by plaque assay in Vero cells. Results are shown as the log of PFU/mg of footpad/ankle joint (*n* = 4).

The data presented showed the protective role of the *AnxA1-FRP2/ALX* pathway in CHIKV infection. Following this, we determined whether exogenous administration of *AnxA1* could attenuate CHIKV disease. Thus, to establish the exogenous effect of *AnxA1* on the course of CHIKV-induced inflammation, WT (BALB/c) mice were either treated or not with the *AnxA1* mimetic peptide (Ac_2–26_ 150µg/animal). Our results showed that prophylactic treatment with Ac_2–26_, administered 1 h before CHIKV infection and at 24, 48 or 72 h post-infection significantly reduced the clinical signs of CHIKV-induced disease. A decrease in the hypernociception parameter occurred from the 3rd dpi (Figure 6G) and a reduction in the formation of paw edema occurred at the 7th dpi (Figure 6H). MPO, CXCL1, and IL-6 levels in the footpad were diminished in the mice treated with Ac_2–26_ compared with untreated animals. In these animals, footpad samples were collected 1 h after the 48-h treatment (therefore at 2 dpi) (Figure 6A–C). However, therapeutic treatment with Ac_2–26_, given as a single dose at 24 h post-infection, was not sufficient to decrease MPO, CXCL1, or IL-6 levels in mice compared with untreated WT (BALB/c) animals (Supplementary Figure S4). Interestingly, consistent with Figure 6A,B, flow cytometry analysis showed that prophylactic treatment with the *AnxA1* mimetic peptide also significantly reduced the number of neutrophils (CD11b^+^Ly6G^+^) in mouse paws compared with untreated WT (BALB/c) animals infected with CHIKV at 2 dpi (Figure 6F), without causing changes in the number of CD45^+^ leukocytes or macrophages (CD11b^+^F4/80^+^) (Figure 6D,E). However, after treatment with Ac_2–26_, there were no consistent or significant differences in paw injury caused by CHIKV, as indicated in the histopathological scores (Figure 6J). Furthermore, the treatment with Ac_2–26_ does not affect the viral titer, as untreated and treated mice showed similar viral loads (Figure 6K). Together, these results demonstrate the antinociceptive and anti-inflammatory effects of exogenous *AnxA1* during CHIKV infection and suggest that its potential to decrease the inflammatory response and clinical signs of virus-induced disease may be related to the duration of clinical signs, treatment cycle, and the peptide dose.

## 4. Discussion

In this study, we investigated the role of the *AnxA1-FPR2/ALX* pathway during CHIKV infection and evaluated the anti-inflammatory and antinociceptive effect of an *AnxA1* mimetic peptide during CHIKV-induced disease. Our main findings were as follows:

(i) Immunostaining of *AnxA1* in the footpad was increased at 7 days after CHIKV infection;
(ii) *AnxA1* deficiency was associated with increased production of inflammatory mediators, enhanced accumulation of neutrophils, exacerbated tissue damage, with minor effects on viral clearance;
(iii) in the absence of the *FPR2* receptor, there was an increase in the production of MPO in the footpad, elevated histopathological damage, and prolonged hypernociception with minimal impact on viral clearance;
(iv) prophylactic treatment with Ac_2–26_ decreased the production of inflammatory mediators in the footpad, reduced neutrophil accumulation in the infection site and resulted in a decrease in paw edema and hypernociception, without interfering with the viral titers of treated mice.

Taken together, these findings demonstrate evidence of the protective role of the *AnxA1*-*FPR2/ALX* signaling axis during CHIKV infection.

We and others have previously described that *AnxA1* is an effective mediator of inflammation resolution involved in various preclinical models of inflammatory diseases such as gout, pleurisy, peritonitis, and arthritis [33,42,43,44,45]. However, *AnxA1* has been less investigated in the context of viral infections [35,46]. Recently we identified a protective role for the *AnxA1-FPR2/ALX* pathway in DENV infection [32]. Several studies have reported a correlation between decreased levels of *AnxA1* in the blood and enhanced disease severity in patients infected with dengue or CHIKV compared with healthy controls [32,34]. However, we showed that plasma levels of *AnxA1* were not altered in mice infected with CHIKV. Despite that, *AnxA1* KO mice were more susceptible to inflammation [36].

Accordingly, in the context of CHIKV infection, *AnxA1* KO mice presented an increase in MPO, CXCL1, and IL-6 levels, alterations that were also observed in patients affected by CHIKV infection [3,4]. Increased production of these inflammatory mediators indicates that the inflammatory response induced by CHIKV was more intense with the deficiency of *AnxA1*. During infection, *AnxA1* KO mice presented an increase in the histopathological score with exacerbation of inflammatory infiltrate and damage to muscle architecture. This could also be associated with increased neutrophil accumulation at the infection site, as indicated by increased immunostaining of CD11b/Ly6G. Associated with that, neutrophils are known to produce high levels of ROS, which are primarily associated with tissue damage in arbovirus infections such as CHIKV [7,8]. Infected macrophages serve as a possible reservoir for CHIKV and may contribute to the persistent inflammation driven by secretion of cytokines (e.g., IL-6, CCL2) and joint pain associated with infection [1,41]. Furthermore, during the inflammatory response, cytokines mediate the recruitment of neutrophils and monocytes/macrophages that are also involved in the production of direct-acting hyperalgesic mediators, such as PGE_2_, leading to mechanical hypernociception and chronic CHIKV in patients [3,10,47,48]. In our study, CHIKV-infected animals showed prolonged hypernociception, even after the inflammation was resolved, indicating that there may be other mechanisms involved in pain persistence.

A hallmark of CHIKV infection is polyarthralgia and polyarthritis, which play a significant role in disease pathogenesis and may intensify patient suffering [3,4,9]. The sustained increase in CCL2 followed by the late increase in TNF suggests that these inflammatory mediators may be related to CHIKV-induced pain persistence. In this regard, it has been shown that TNF and activation of dorsal root ganglion (DRG)-resident macrophages are associated with persistent nociception even when the inflammation is resolved [49]. Interestingly, our group reported the TNF role in the persistent pain phenotype in CHIKV-infected mice, showing the reliability of our experimental model of chikungunya fever seen in humans (unpublished data). At the same time, our results corroborate previous findings, which showed that during CHIKV infection, there is a significant increase in levels of inflammatory mediators, paw swelling, and tissue damage [50,51].

*AnxA1* acts through binding to the receptor *FPR2* [28,30]. Here, we demonstrate that the absence of *FPR2* during CHIKV infection resulted in a phenotype of exacerbated acute inflammation with minimal impact on viral clearance, similar to that observed in *AnxA1* KO mice. Moreover, the *Fpr2/3* KO mice showed a prolonged increase in MPO levels in the footpad compared with their uninfected control (mock *Fpr2/3* KO). Similar findings were demonstrated in a murine model of pneumococcal pneumonia, in which *Fpr2/3* KO mice also had shown exacerbated inflammatory response compared with their WT (C57BL/6) counterparts [29]. This effect could, at least in part, be explained by the fact that the *FPR2* receptor also binds and is activated by other pro-resolving mediators, including resolvin D1 and lipoxin A4 [26]. Future research should be conducted, in order to evaluate the role of other *FPR2* agonists during CHIKV infection.

Given the results obtained with the *AnxA1* KO or *Fpr2/3* KO mice, we focused on analyzing the pharmacological effect of an *AnxA1* mimetic peptide, Ac_2–26_. Previous studies demonstrated that the treatment with pro-resolving agonists has beneficial effects on infectious diseases, including influenza, dengue, and bacterial pneumonia [29,32,46].

In our study we show that the treatment with Ac_2–26_, one hour before the CHIKV infection and every 24 h for the next 3 three days, reduced the levels of CXCL1 and decreased the neutrophil count in the paw of CHIKV-infected mice, without interfering with the viral titers at the site of infection (paw/ankle joint) and independently of viral infectivity. Moreover, Ac_2–26_ decreased IL-6 levels in the footpad, a cytokine widely related to persistent pain in CHIKV-infected patients [3,10]. These results could be associated with the decreased paw edema and hypernociception observed in mice treated with Ac_2–26_. Supporting our findings, it has been described that Ac_2–26_ mediates an anti-nociceptive effect through *FPR2/ALX* and increases *AnxA1* expression in rat DRG [52]. Furthermore, it has been described that Ac_2–26_ reduces hyperalgesia by decreasing neutrophil accumulation and reducing IL-1β and CXCL1 production in the periarticular tissue [43].

A key to the development of safe therapeutic interventions is reducing inflammation without compromising the hosts ability to control viral replication [18]. Interestingly, mice with *AnxA1* or *Fpr2/3* deficiency had little effect on recovered viral loads upon CHIKV inoculation. Similar findings were observed in a model of DENV infection (Costa et al., 2022). However, Arora and colleagues have demonstrated in a murine model of influenza A that *AnxA1* KO mice showed an increased viral clearance and accumulation of neutrophils at the infection site [46]. Indeed, *AnxA1*-deficient neutrophils are known to be more responsive to inflammatory stimuli [53]. In addition, neutrophils recruited to the infection site produce ROS and NET, neutralizing the CHIKV [7]. Thus, future studies to elucidate the pathways regulating *AnxA1* expression in neutrophils in the context of CHIKV infection are crucial to identifying new therapeutic strategies.

In conclusion, the findings reported here demonstrate that endogenous *AnxA1* plays a fundamental role in controlling the production of inflammatory mediators and accumulation of neutrophils during CHIKV infection. Furthermore, we showed that treatment with the Ac_2–26_ peptide reduces the main clinical signs of disease caused by CHIKV, such as edema and hypernociception. These results reinforce the idea that modulating the excessive inflammation early in the infection is crucial for adequate resolution. Therefore, improvement of endogenous *AnxA1* or exogenous administration of its peptidomimetic Ac_2–26_ may represent a powerful anti-inflammatory strategy against the host’s excessive immune response induced by CHIKV. These strategies have been shown to be effective in several inflammatory and infectious conditions including sterile articular inflammation [30,42,43], bacterial pneumonia [29], and more recently, viral infections such as dengue [32] and Zika [35]. Therefore, our findings suggest that targeting the *AnxA1-FPR2/ALX* pathway might be a therapeutic strategy for controlling excessive acute inflammation and hypernocyception induced by CHIKV without affecting the host’s ability to deal with the virus.

## Data Availability

The data presented in this study are available on request from the corresponding author.

## Supplementary Materials

The following supporting information can be downloaded at: https://www.mdpi.com/article/10.3390/cells11172717/s1, Figure S1: Immune cell profile in the paw and popliteal lymph node of *AnxA1* or WT CHIKV-infected mice, Figure S2: Absence of Formyl Peptide Receptor (*FPR2*) increases MPO levels upon CHIKV infection, Figure S3: Mechanical hypernociception and paw edema in mice infected with CHIKV, Figure S4: Effect of Ac_2–26_ therapeutic treatment in the inflammatory response during CHIKV infection.
