# Supplementary material for: Annexin A1-FPR2/ALX Signaling Axis Regulates Acute Inflammation during Chikungunya Virus Infection
Source: Cells. 2022 Aug 31;11(17):2717. doi: 10.3390/cells11172717 (PMC9454528; doi:10.3390/cells11172717)
Supplement: Supplementary file 1 [file cells-11-02717-s001.zip › cells-1876556-supplementary.pdf]

Supplementary Materials:

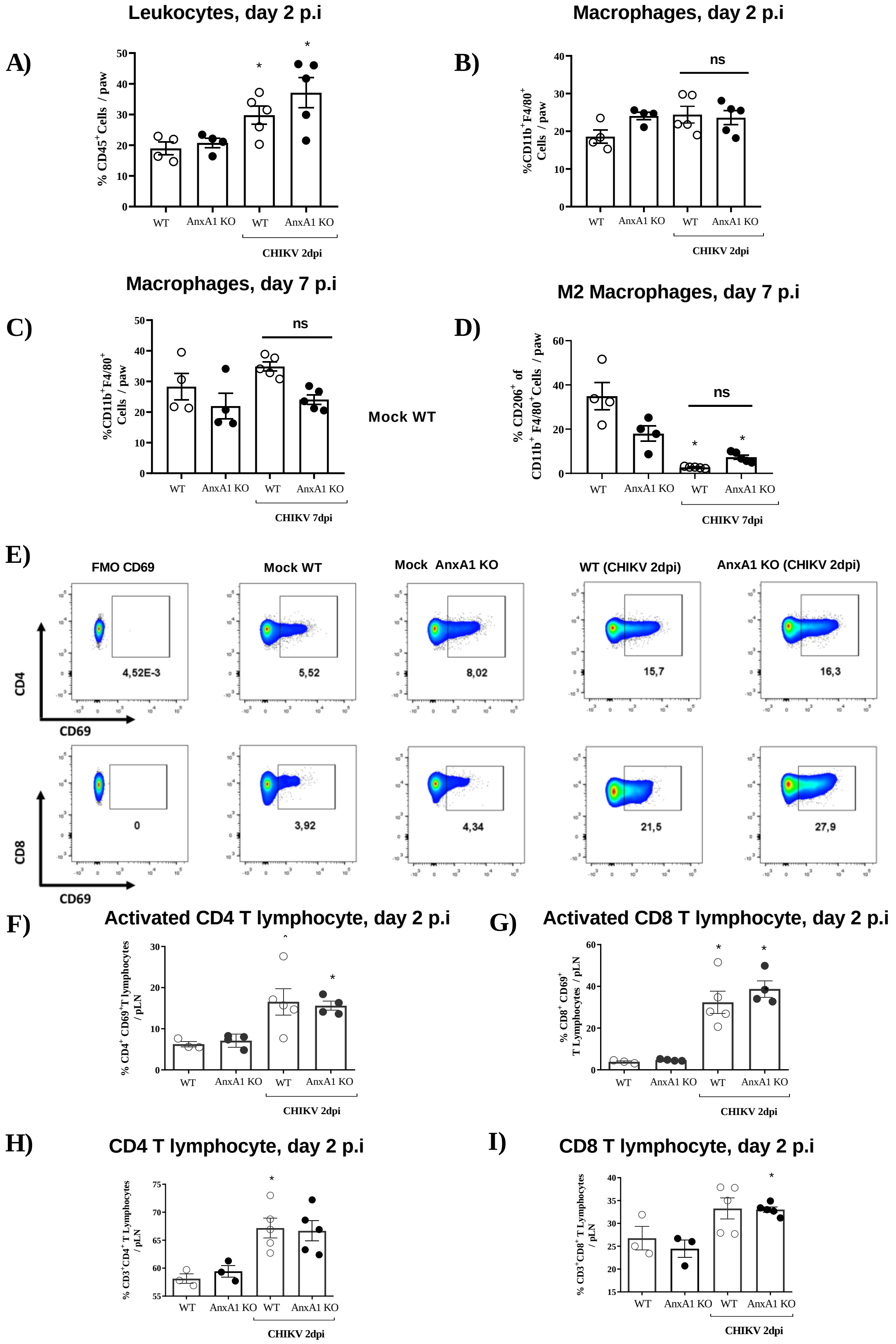

Supplementary Figure S1. Immune cell profile in the paw and popliteal lymph node of AnxA1 or WT CHIKV-infected mice. (A) Percentage of CD45+ cells, macrophages (B and C), M2 macrophages (D), per footpad at 2 or 7 dpi. Activated CD4+ T cells (F), activated CD8+ T cells (G), CD4+ T cells (H),

and CD8+ T cells (I) per popliteal lymph node (pLN) in mock (n = 5), and AnxA1 KO or WT CHIKV-infected mice on 2 dpi. (E) dot plot showing the T cell activation profile. The dot plot demonstrates that the data were analyzed from the "Cell FMO limits" and all fluorescence values above this value were considered true positives. Statistical significance was calculated using one-way ANOVA with Tukey comparisons test. Data are presented as mean ± SEM (n = 4-5 per group); *$p < 0.05$. when compared to the uninfected control (mock).

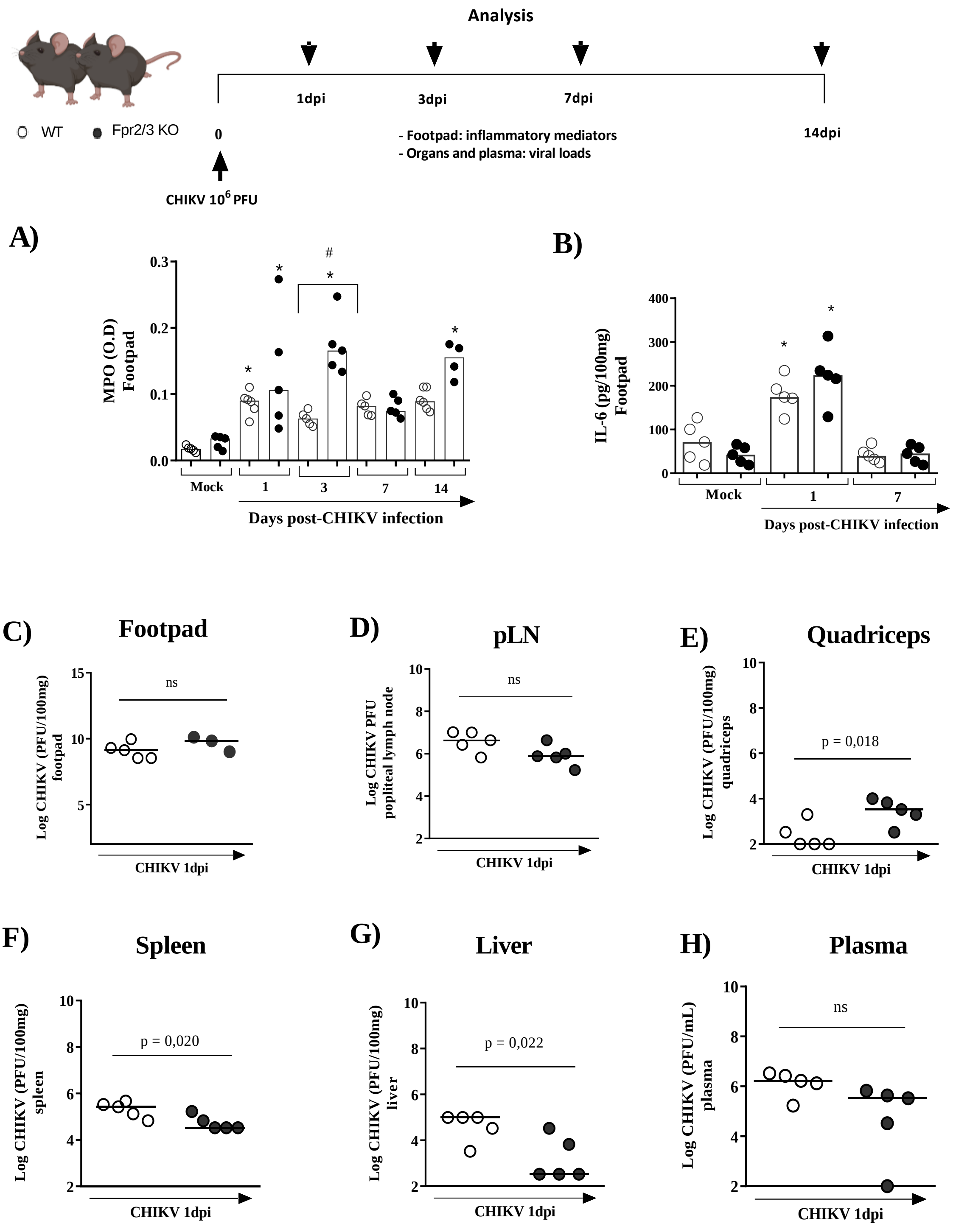

Supplementary Figure S2. Absence of Formyl Peptide Receptor (FPR2) increases MPO levels upon CHIKV infection. Fpr2/3 KO or WT (C57BL6) mice were infected through the intraplantar (i.pl.) route with CHIKV 10^6 PFU and were euthanized at 1,3,7 or 14 days post-infection. A) MPO, and B) IL-6

levels in the footpad. (C-H) Viral load in the footpad, pLN, quadriceps, spleen, liver, and plasma of infected mice. Statistical analysis was performed using the two-way ANOVA with Sidak comparisons test. Results are shown as the mean ± SEM, and there was n=5-6 animals in each group. * p<0.05 when compared to control uninfected mice (mock) or #p<0.05 when compared Fpr2/3 KO and WT infected mice. For quantification of infectious virus loads, the analysis was performed using the unpaired, T-test.

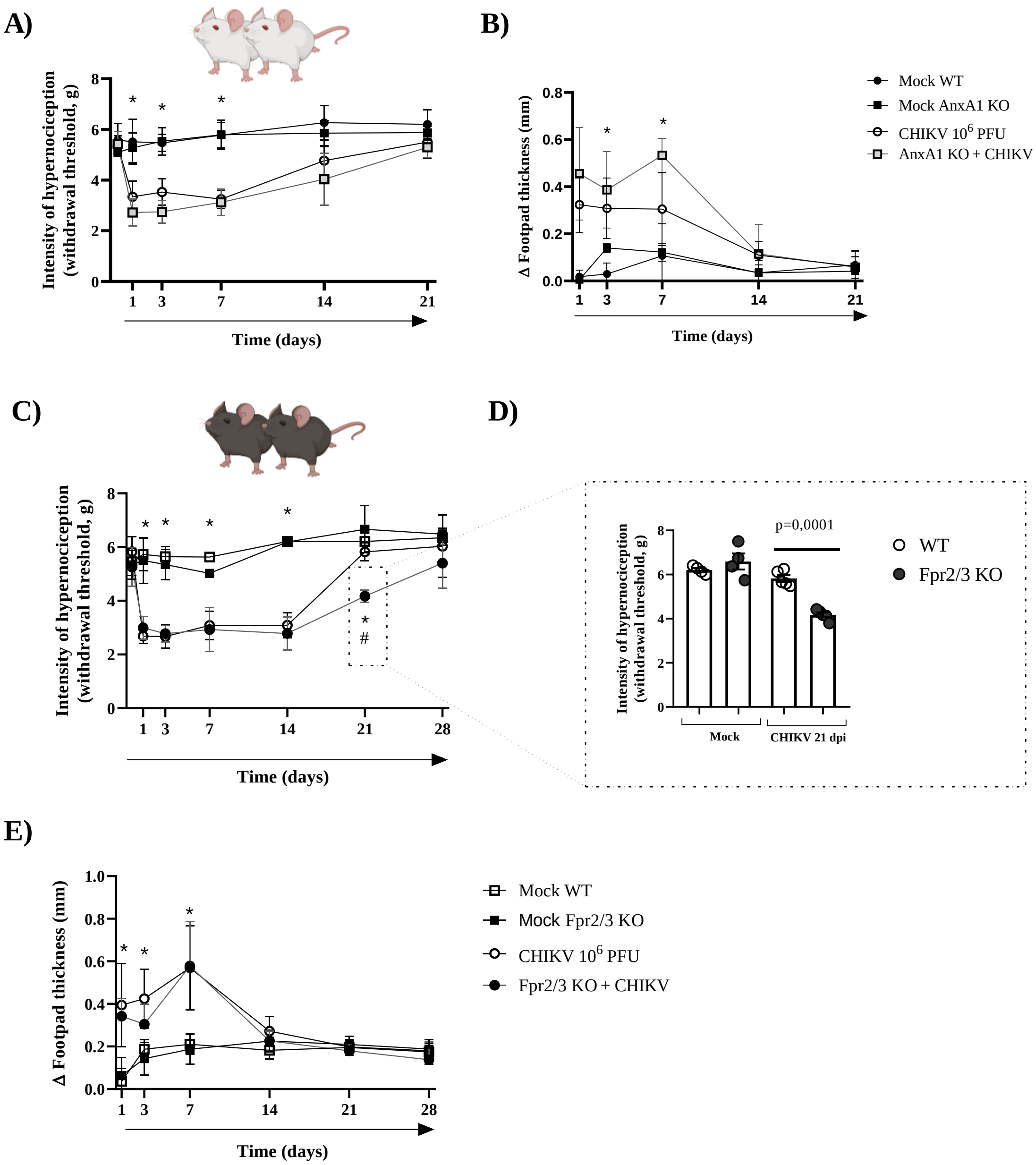

Supplementary Figure S3. Mechanical hypernociception and paw edema in mice infected with CHIKV. AnxA1 KO or WT (BALB/c), and Fpr2/3 KO or WT (C57BL6) mice were infected with CHIKV

(10⁶ PFU / 30µL, i.pl.). The animals were monitored for 21 or 28 days for parameters of (A, C, and D) Mechanical hypernociception and (B and E) Paw swelling. Data for mechanical hypernociception is shown as the force (g) required to induce dorsiflexion of the tibiotarsal joint, followed by paw withdrawal. The results for paw edema were expressed as the Δ of the difference between the baseline measurement and the post-infection measurement, expressed in mm. Statistical significance was calculated using two-way ANOVA with Sidak comparisons test. Data are presented as mean ± SEM (n = 5 - 6 per group); *p < 0.05 when compared to the uninfected control (mock).

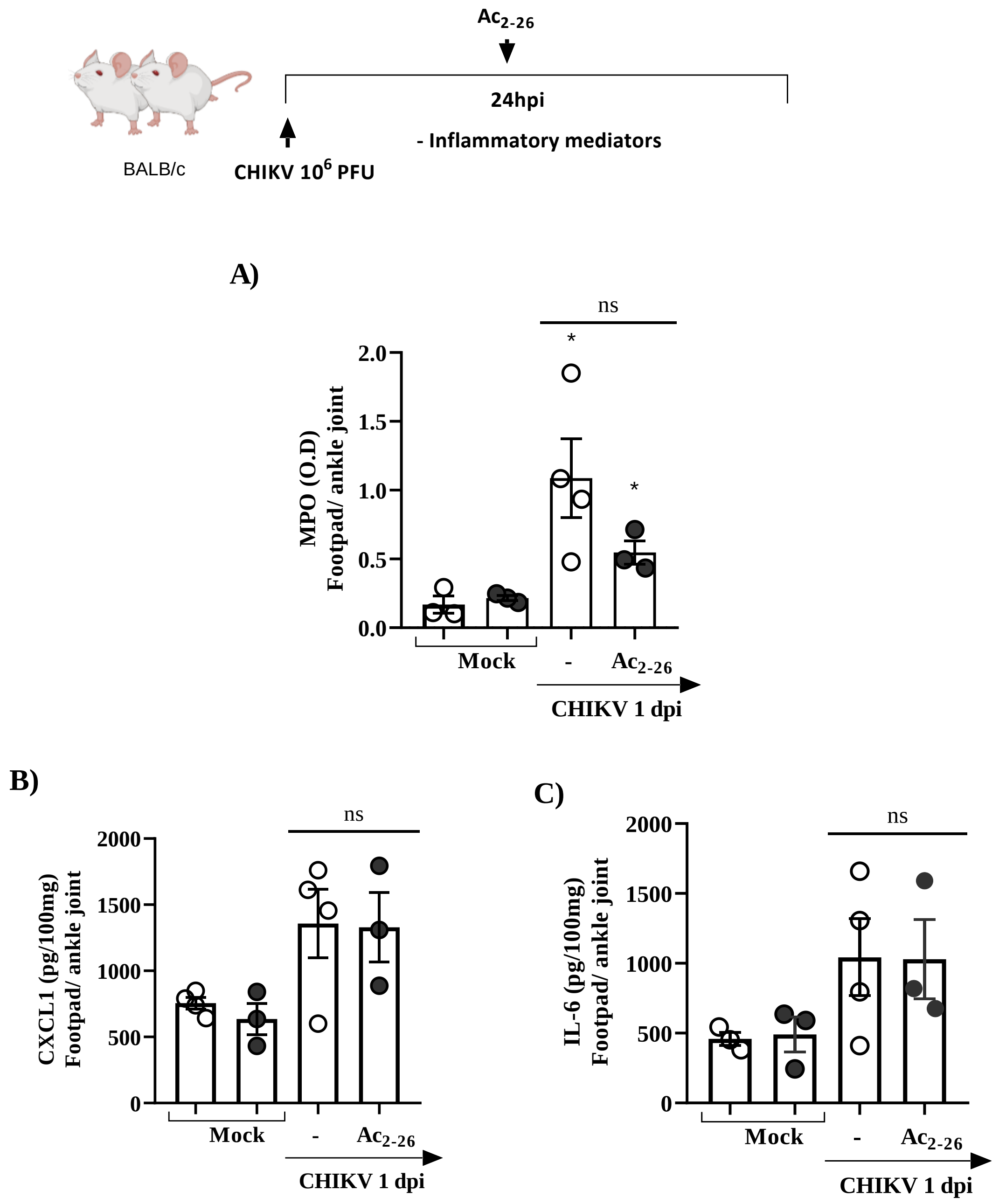

Supplementary Figure S4. Effect of Ac_{2-26} therapeutic treatment in the inflammatory response during CHIKV infection. Ac_{2-26} treatment (150 µg/mice, intraperitoneal route) was administered as a single dose 24 hours post-infection with CHIKV (10^6 PFU / 30µL, i.pl.). At 1 h after the treatment, the footpad tissue was collected. (A) MPO, (B) CXCL1, and (C) IL-6 levels in the footpad / ankle joint. Statistical

significance was calculated using one-way ANOVA with Tukey post-test. Data are presented as mean ± SEM ($n = 5$ per group), *$p < 0.05$, ns - not significant.
